# Requirements for human cardiomyocytes

**DOI:** 10.1111/cpr.13150

**Published:** 2021-10-27

**Authors:** Miao Yu, Wei Lei, Jiani Cao, Lei Wang, Aijin Ma, Zhen‐Ao Zhao, Huang‐Tian Yang, Zhenya Shen, Feng Lan, Feng Cao, Ping Liang, Xuetao Pei, Andy Peng Xiang, Junying Yu, Yu Zhang, Yong Zhang, Qiyuan Li, Jiaxi Zhou, Jun Wei, Yaojin Peng, Huanxin Zhu, Lingmin Liang, Nan Cao, Boqiang Fu, Jie Hao, Tongbiao Zhao, Shijun Hu

**Affiliations:** ^1^ Department of Cardiovascular Surgery of The First Affiliated Hospital & Institute for Cardiovascular Science Collaborative Innovation Center of Hematology State Key Laboratory of Radiation Medicine and Protection Medical College Soochow University Suzhou China; ^2^ National Stem Cell Resource Center State Key Laboratory of Stem Cell and Reproductive Biology Institute of Zoology Institute for Stem Cell and Regeneration University of Chinese Academy of Sciences Beijing China; ^3^ Beijing Institute for Stem Cell and Regenerative Medicine Beijing China; ^4^ Chinese Society for Stem Cell Research Shanghai China; ^5^ Beijing Technology and Business University Beijing China; ^6^ Shanghai Institute of Nutrition and Health Chinese Academy of Sciences Shanghai China; ^7^ State Key Laboratory of Cardiovascular Disease National Center for Cardiovascular Diseases Key Laboratory of Application of Pluripotent Stem Cells in Heart Regeneration Fuwai Hospital Chinese Academy of Medical Sciences and Peking Union Medical College Beijing China; ^8^ The Second Medical Center Chinese PLA General Hospital National Clinical Research Center for Geriatric Disease Beijing China; ^9^ Institute of Translational Medicine Zhejiang University Hangzhou China; ^10^ South China Cell & Stem Cell Bank SCIB Guangzhou China; ^11^ Center for Stem Cell Biology and Tissue Engineering Key Laboratory for Stem Cells and Tissue Engineering Ministry of Education Sun Yat‐Sen University Guangzhou China; ^12^ Nuwacell Biotechnologies Co., Ltd Hefei China; ^13^ Zephyrm Biotechnologies Co., Ltd Beijing China; ^14^ National Clinical Research Center of Respiratory Disease First Affiliated Hospital of Guangzhou Medical University Guangzhou China; ^15^ HHLIFE Company Inc. Shenzhen China; ^16^ China National GeneBank Shenzhen China; ^17^ Institute of Hematology and Blood Diseases Hospital Chinese Academy of Medical Sciences & Peking Union Medical College Tianjin China; ^18^ National Institute of Metrology Beijing China

**Keywords:** human cardiomyocytes, quality control, standard

## Abstract

‘Requirements for human cardiomyocytes', jointly drafted and agreed upon by experts from the Chinese Society for Stem Cell Research, is the first guideline for human cardiomyocytes in China. This standard specifies the technical requirements, test methods, test regulations, instructions for use, labelling requirements, packing requirements, storage requirements, transportation requirements and waste disposal requirements for human cardiomyocytes, which is designed to normalize and standardize human cardiomyocyte research and production. It was originally released by the China Society for Cell Biology on 9 January 2021. We hope that the publication of this guideline will promote institutional establishment, acceptance and execution of proper protocols, and accelerate the international standardization of human cardiomyocytes for applications.

## 1 SCOPE

This document specifies the technical requirements, test methods, test regulations, instructions for use, labelling requirements, packing requirements, storage requirements, transportation requirements and waste disposal requirements for human cardiomyocytes. This standard is applicable for the quality control of human cardiomyocytes.

## 2 NORMATIVE REFERENCES

The following content constitutes indispensable articles of this standard through normative reference. For dated references, only the edition cited applies. For undated references, only the latest edition (including all amendments) applies.

GB/T 6682 *Water for analytical laboratory use‐specification and test methods*

WS 213 *Diagnosis for hepatitis C*

WS 273 *Diagnosis for syphilis*

WS 293 *Diagnosis for HIV/AIDS*

*Pharmacopoeia* *of the People's Republic of China*

*National* *Guide to Clinical Laboratory Procedures*

T/CSCB 0001‐2020 *General requirements for stem cells*

T/CSCB 0002‐2020 *Requirements for human embryonic stem cells*

## 3 TERMS AND DEFINITIONS

The following terms and definitions apply to this document.

### 3.1 Human cardiomyocytes

Cardiac muscle cells with excitability, conductivity, autorhythmicity and contractility, which is particularly rich in myofibril, striae and mitochondria.

### 3.2 Membrane potential

The potential difference between the inside and outside of the cell caused by ion migration, which is determined by concentration gradients of ions across the membrane or by membrane permeability to each type of ion.

### 3.3 Field potential

Changes in extracellular local potential in the formation of synchronous discharge of cell population.

### 3.4 Calcium transient

Rapid oscillation of cytoplasmic calcium concentration caused by action potential or other reasons.

## 4 ABBREVIATIONS

The following abbreviations are applicable for this document.

EBV: Epstein‐Barr virus

HBV: hepatitis B virus

HCMV: human cytomegalovirus

HCV: hepatitis C virus

HIV: human immunodeficiency virus

HTLV: human T‐lymphotropic virus

STR: short tandem repeat

TP: treponema pallidum

## 5 TECHNICAL REQUIREMENTS

### 5.1 Raw materials and auxiliary materials

#### 5.1.1 For the harvesting of human biological raw materials, the domestic laws and ethical regulations shall be complied.

#### 5.1.2 The raw materials, reagents, consumables, and other auxiliary materials and/or supplies (eg gases) shall meet the requirements of T/CSCB 0001‐2020 and T/CSCB 0002‐2020.

#### 5.1.3 The donor shall be negative for HIV, HBV, HCV, HTLV and TP.

### 5.2 Primary quality attributes

#### 5.2.1 Cell morphology

With two‐dimensional culture, human cardiomyocytes shall be adherent growth and exhibit spindle‐shaped, short column‐shaped or irregular shaped. Most of them shall be mononuclear, while some of them can be coenocytic occasionally.

#### 5.2.2 Cell viability

≥90% before cryopreservation and ≥60% after resuscitation.

#### 5.2.3 Cell markers

cTnT‐positive rate ≥70.0% and α‐actinin‐positive rate ≥70.0%.

#### 5.2.4 Membrane potential or field potential

Shall exhibit typical waveform.

#### 5.2.5 Calcium flux

Shall exhibit periodic variation of calcium signal.

#### 5.2.6 Contractility

Shall exhibit rhythmical mechanical contraction spontaneously or induced.

#### 5.2.7 Pharmacological action

The beat frequency shall elevate with isoproterenol treatment while decline when treated with carbachol.

#### 5.2.8 Microorganisms

Shall be negative for fungi, bacteria, mycoplasma, HIV, HBV, HCV, HTLV, EBV, HCMV and TP.

### 5.3 Process control

The process of cell expansion, differentiation, cryopreservation and resuscitation shall follow the requirements of T/CSCB 0001‐2020. Cell STR shall be consistent with the donor’s identity.

## 6 TEST METHODS

### 6.1 Cell morphology

Observe the morphology of cells grown in 2D condition under microscope.

### 6.2 Cell viability

The method in Appendix A shall be followed.

### 6.3 Cell markers

The method in Appendix B shall be followed.

### 6.4 Membrane potential

The method in Appendix C shall be followed.

### 6.5 Field potential

The method in Appendix D shall be followed.

### 6.6 Calcium flux

The method in Appendix E shall be followed.

### 6.7 Contractility

Observe the contractility under microscope.

### 6.8 Drug reactivity

Observe the contractility under microscope under the treatment of isoproterenol (1 µM) or carbachol (1 µM) and count beats per minute.

### 6.9 Microorganisms

The method for microbiological detection referred in T/CSCB 0001‐2020 shall be followed.

## 7 INSPECTION RULES

### 7.1 Sampling method

1. Cells produced from the same production cycle, same production line, same source, same passage and same method are considered to be the same batch.
2. Three smallest units of packaging shall be randomly sampled from the same batch.

### 7.2 Quality inspection and release

1. Each batch of products shall be subject to qualify inspection before release, and inspection reports shall be attached.
2. The quality inspection items shall include all the attributes specified in 5.2.

### 7.3 Review inspection

Review inspection shall be performed by professional cytological testing institutions or laboratories as necessary.

### 7.4 Decision rules

1. Products that pass all requirements in 5.2 for the quality inspection for release are considered to be qualified. Products that fail to pass one or more requirements in 5.2 for the quality inspection for release are considered to be unqualified.
2. Products that pass all requirements in 5.2 for the quality review inspection are considered to be qualified. Products that fail to pass one or more requirements in 5.2 for the review inspection are considered to be unqualified.

## 8 INSTRUCTION FOR USAGE

The instructions for usage shall include, but not limited to

1. Product name;
2. Passage number;
3. Cell number;
4. Production date;
5. Lot number;
6. Production organization;
7. Storage conditions;
8. Shipping conditions;
9. Contact information;
10. Operation manual;
11. Execution standard number;
12. Manufacturing address;
13. Postal code;
14. Matters that deserve attention.

Note: indicate endotoxin content if required

## 9 LABELS

The label shall include but not limited to

1. Product name;
2. Passage number;
3. Cell number;
4. Lot number;
5. Production organization;
6. Production date.

## 10 PACKAGE, STORAGE AND TRANSPORTATION

### 10.1 Package

The appropriate materials and containers shall be selected to ensure maintenance of the primary quality attributes of human cardiomyocytes.

### 10.2 Storage

1. T/CSCB 0001‐2020 shall be followed.
2. Productions should be stored in liquid nitrogen.

### 10.3 Transportation

1. T/CSCB 0001‐2020 shall be followed.
2. Cryopreserved cell products shall be transported in dry ice or liquid nitrogen.

## 11 WASTE DISPOSAL

T/CSCB 0001‐2020 shall be followed.

## CONFLICT OF INTEREST

No potential conflicts of interest are disclosed.

## AUTHOR CONTRIBUTIONS

SH, TZ, JH and BF contributed to conception and design. MY, WL, JC and LW drafted and revised the manuscript. AM, ZZ, HY, ZS, FL, FC, PL, XP, PX, JY, YZ, YZ, QL, JZ, JW, YP, HZ, LL and NC critically read and revised the manuscript.

## Figures and Tables

**FIGURE A1:**
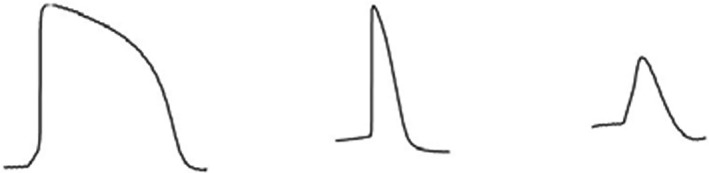
Three general types of action potential for human cardiomyocytes

**FIGURE A2:**
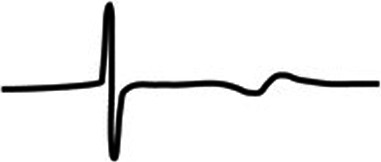
Typical waveform of field potential for human cardiomyocytes

**FIGURE A3:**
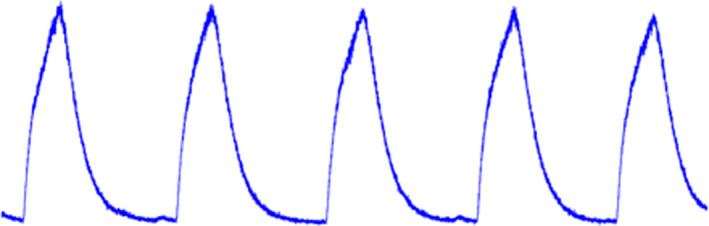
Periodic variation of calcium signal for human cardiomyocytes

## Data Availability

n/a.

## APPENDIX A

(Normative appendix)

Cell viability test (cell enumeration method)

### A.2 Reagents

Unless otherwise stated, all reagents used shall be analytical grade. The water used for testing shall be deionized water.

### A.3 Test protocol

#### A.3.1 Preparation of cell suspension

Harvest and suspend the cells with appropriate volume of PBS (A.2.1). The cell density shall be 20‐50 cells/mm^2^. Serial dilution is necessary if the number of cells exceeds 200 per square.

#### A.3.2 Trypan blue staining

Evenly mix the trypan blue solution (A.2.2) with the cell suspension (A.3.1) at a volume ratio of 1:1.

#### A.3.3 Cell counting

Load the haemocytometer (A.1.2) with 10 μl of the trypan blue‐labelled sample (A.3.2). Make sure the entire chamber is filled with the testing sample. Stand for 30 seconds, count the stained cells and the total number of cells respectively under microscope (A.1.1).

For the 16 × 25 counting chamber, use the four 1 mm^2^ medium squares at the top left, top right, bottom left and bottom right of the chamber (ie 100 small squares) for counting. For the 25 × 16 counting chamber, use the five 1 mm^2^ medium squares at the top left, top right, bottom left, bottom right and centre of the chamber (ie 80 small squares) for counting.

When there are cells on the lines of the large square, only cells on the top line and left line of the large square can be counted (or alternatively only cells on the bottom line and right line).

### A.4 Calculation and analysis

Cell viability is calculated according to equation (1):

$$S=\left(M‐D\right)/M\times 100\%.\left(1\right)$$

In the equation:

S—viability of cells

M—total number of cells

D—number of stained cells

The viability of cells is the mean of two duplicate samples. Two independent cell viability tests shall be performed on the same sample. The mean value of two independent viability tests is recorded as the viability of cells.

### A.5 Accuracy

The absolute difference value between the two independent tests, under the same conditions, shall not exceed 10% of their arithmetic mean.

## APPENDIX B

(Normative appendix)

Detection of cell markers (flow cytometry)

### B.2 Reagents

Unless otherwise stated, all the reagents used shall be analytical grade. The water used in the experiment shall be Grade 1 water as stipulated in GB/T 6682.

### B.3 Sample storage

The wash solution and fixed samples shall be stored at 2‐8°C. The fixing solutions shall be aliquoted, sealed, labelled and stored at or below –20°C. Antibodies shall be stored according to the manufacturer's instructions.

### B.4 Test protocol

#### B.4.1 Sample preparation and fixation

Collect samples by centrifuging single‐cell suspensions at 250 g for 3 minutes. Discard the supernatant. Resuspend the cells in an appropriate volume of fixing solution and incubate for 10 minutes in an ice bath. Wash the cell samples with an appropriate volume of wash solution for 3‐5 times (3‐5 minutes each time).

#### B.4.2 Blocking and permeabilization

Resuspend the fixed sample (B.4.1) with the blocking/permeabilization solution and aliquot the cells into two independent samples, which will be used as a testing sample and an isotype control sample respectively. Incubate the samples on ice for 20 minutes and then wash the samples with the wash solution.

#### B.4.3 Antibody incubation

Incubate the samples with the diluted antibodies or corresponding isotype controls according to the manufacturer's instructions.

#### B.4.4 Filtering and loading

Resuspend the samples with wash solution and then transfer the cell suspension into flow cytometry tube by filtering the samples through a mesh with 40 μm pores. Load the samples into the flow cytometer and perform testing according to the manufacturer's instruction.

#### B.4.5 Gating

Gate the target population of cells based on particle size and transparency, excluding cell debris and other irrelevant particles. The gating of positive staining cells shall be determined by the fluorescence intensity using isotype controls as a reference. Both positive and negative experimental controls shall be set up for gating and the following analysis.

### B.5 Analysis of results

Analyse the results using software according to manufacturer's instructions.

## APPENDIX C

(Normative appendix)

Detection of membrane potential (patch clamp)

### C.2 Reagents

Unless otherwise stated, all the reagents used shall be analytical grade. The water used in the experiment shall be Grade 1 water as stipulated in GB/T 6682.

### C.3 Test protocol

#### C.3.2 Microelectrode preparation

Pull the glass capillary tube with micropipette puller (C.1.2) to form a pipette tip with diameter around 1‐2 mm. Daub the pipette tip with silicone resin (C.2.1) and polish it with polishing instrument (C.1.3).

#### C.3.3 Patch‐clamp conduction

Use the manipulator to touch the cell membrane with the pipette tip. In whole‐cell measurements, changes in membrane potential shall be observed in current‐clamp mode.

### C.4 Data acquisition and analysis

With software integrated in patch‐clamp experimental system, action potentials shall be recorded. Three general types of cardiac action potential are presented in Figure A1, which shall be divided into depolarization and repolarization. Time in millisecond (ms) shall be on the *X*‐axis and membrane potential in millivolts (mV) shall be on the *Y*‐axis.

## APPENDIX D

(Normative appendix)

Detection of field potential (microelectrode arrays)

### D.3 Test protocol

#### D.3.1 Cell preparation

After cell enumeration followed by Appendix A, appropriate cell shall be seeded into microelectrode arrays cell culture plate (D.1.3) and sealed by water.

#### D.3.2 Microelectrode arrays conduction

Record the field potential of human cardiomyocytes by microelectrode arrays testing system (D.1.2).

### D.4 Data acquisition and analysis

Typical waveform of cardiac field potential is presented in Figure A2. Time in millisecond (ms) shall be on the X‐axis and field potential in microvolts (mV) shall be on the Y‐axis.

## APPENDIX E

(Normative appendix)

Detection of calcium transient (calcium staining)

### E.2 Reagents

Unless otherwise stated, all the reagents used shall be analytical grade. The water used in the experiment shall be Grade 1 water as stipulated in GB/T 6682.

### E.3 Test protocol

#### E.3.2 Fluo‐AM incubation

Incubate the cell with Fluo‐AM (E.2.1) according to the manufacture’s instruction.

#### E.3.3 Fluorescence detection

Record the fluorescence signal under laser confocal microscope (E.1.3). An excitation wavelength of 480 – 500 nm and an emission wavelength of 525 – 530 nm were adopted.

### E.4 Data acquisition and analysis

Periodic variation of calcium signal is presented in Figure A3. Time in millisecond (ms) shall be on the *X*‐axis and standard fluorescence intensity in F/F0 shall be on the *Y*‐axis, F0 represents resting fluorescence intensity.

